# Music Student’s Approach to the Forced Use of Remote Performance Assessments

**DOI:** 10.3389/fpsyg.2021.641667

**Published:** 2021-04-15

**Authors:** Laura Ritchie, Benjamin T. Sharpe

**Affiliations:** ^1^University of Chichester Conservatoire, University of Chichester, Chichester, United Kingdom; ^2^Institute of Sport, University of Chichester, Chichester, United Kingdom

**Keywords:** resilience, self-efficacy, higher education, assessment, COVID19, music

## Abstract

Music students at the University of Chichester Conservatoire completed questionnaires about their experience of the forced use of remote teaching and learning due to Lockdown, as imposed in the United Kingdom from March to June 2020, and how this impacted their self-beliefs, decision making processes, and methods of preparation for their performance assessments. Students had the choice to either have musical performance assessed in line with originally published deadlines (still in Lockdown) via self-recorded video or defer the assessment until the following academic year. Student’s choice to defer or submit the assessment during Lockdown was influenced by a range of forced factors, such as adaptions required by online teaching, limitations of rehearsal in their home environment, and the challenges in facilitating and recording their own assessments. Students completed online questionnaires about their self-efficacy, resilience, wellbeing, and provided free text responses explaining the reasoning for their decision to record their performance or to defer the assessment were coded to reveal patterns impacting their decision and preparation processes. Those choosing to submit their assessments demonstrated more strategies in their preparation and reported higher perceived self-efficacy scores. The specific conditions for this assessment, as a result of Lockdown, revealed correlations between resilience and both self-efficacy and wellbeing. The impact on teaching and the student experience is discussed and suggestions to support students in future settings of blended delivery are presented. Theoretical and practical implications are discussed.

## Introduction

Musicians transmit their art to listeners through performance. Within music education, students develop their skills in performance and are assessed as they develop and progress through their degree programs. These performance assessments are affected by a range of contextual and situational influences, such as the physical properties of the hall or space and interactions with others in the performance setting, even before the interpretative and evaluative judgments are considered (McPherson and Thompson, 1998). Beside the skill, expertise, and associated physical demands required to execute a successful performance, the process of preparing for a musical performance assessment involve various qualities related to a person’s self, such as resilience, self-efficacy, managing wellbeing, and the use of self-regulated learning strategies (Ericsson et al., 1993; Williamon, 2004; Miksza, 2012; Ritchie and Williamon, 2012; McPherson et al., 2019).

The novel coronavirus (SARS-CoV-2), and the forced quarantine of Lockdown has had a significant impact globally (Brooks et al., 2020; Jiang, 2020; Petzold et al., 2020; Qiu et al., 2020; Remuzzi and Remuzzi, 2020; Ritchie et al., 2020; Walsh, 2020; Yıldırım and Güler, 2020). The higher education sector has not been immune to the disruption and has met the situation by quickly and dramatically adjusting aspects of learning, teaching, and assessment. Music students in higher education have been impacted by the move to online education during this pandemic, specifically in terms of their interpersonal relationships with their teachers, and in general, by reporting more stress and fewer dedicated practice hours (Philippe et al., 2020; Rosset et al., 2021). Although in recent decades student learning processes have been more actively integrated into the assessment process, the range of assessment strategies within the higher education sector is not diverse (Boud and Falchikov, 2006; Craddock and Mathias, 2009). Change has been seen in higher education assessment practices, an example of which can be found in Florence, where alternate practices of peer and self-assessment were adopted to engage students as active agents in their learning (Di Stasio et al., 2019).

The UK Quality Assurance Agency (QAA, 2018) outlines guiding principles for assessment in higher education which include advice that assessment is “explicit and transparent” and “students are supported and prepared for assessment” (p. 5–6). In times of a pandemic, this is a challenge, especially when everything from the mode of delivery, to the location, to the timing, as well as aspects of the assessment itself changes in a matter of days.

The student experience is wider than the assessment context, and aspects of mental health and wellbeing have gained attention in education globally, with students showing increasing signs of stress (Russell and Topham, 2012; Macaskill, 2013; Hamdan-Mansour et al., 2014). Universities are becoming increasingly aware of the importance of understanding student mental health and wellbeing and employing preventative measures to alleviate this increasing problem (Dehaas, 2011). As places of learning, institutions continually work to improve students’ experience in education, fostering resilience in learning, and preparing students for their professional lives after graduation (Spronken-Smith, 2013).

The understanding of resilience as a multidimensional mechanism combining several personal characteristics and skills, internal processes, and external outcomes (Leipold and Greve, 2009; Goodenough et al., 2020) has evolved. It has moved from focusing solely on having the psychological strength to maintain stability during times of stress and change (Flach, 1997; Combes-Malcome, 2007), to include a much broader definition including the importance of “accessing and maintaining wellbeing and wider interactions with family, community, and culture” as part of the response to an adverse situation (Ungar, 2008, p. 225). Pooley and Cohen (2010) went on to define resilience holistically as “the potential to exhibit resourcefulness by using available internal and external resources in response to different contextual and developmental challenges” (p. 30). Independent of the definition adopted, the adversity (change or risk) and successful navigation or adaption to a presenting challenge are fundamentally at the core of resilience in research settings (Schilling, 2008). In music, the relationship of resilience has received less attention, but is no less important. Kegelaers et al. (2020) found an inverse relationship between mental health and resilience and noted that music students were less resilient and more symptomatic than professionals. Osborne et al. (2014) suggest resilience training can enhance the wellbeing of music students in higher education.

Factors that relate positively to resilience are considered protective factors, and these come into play when dealing with the risk or adversity aspect involved in a conflict or challenging situation. For example, Lightsey (2006) theorized that a strong sense of self-efficacy was important for maintaining high levels of resilience. Self-efficacy beliefs are the self-beliefs people hold regarding their capabilities to carry out specific tasks and this criterial self-belief would then be a protective factor relating to resilience. Bandura (1977, 1993) first introduced and investigated self-efficacy beliefs and practically investigated the construct’s strength by testing people’s beliefs in their capabilities to carry out tasks relating to their phobias. This initial testing was in a psychotherapy setting, and these beliefs have since been found to be relevant to many areas of life within disciplines studied in higher education, from academic pursuits, to sport, to music (Pajares, 1996; Feltz et al., 2008; Ritchie and Williamon, 2011) and in wider areas of everyday functioning such as self-care (D’Souza et al., 2017). People’s self-efficacy beliefs affect their choice of task, persistence, and perseverance, which all impact their achieved outcomes (Zimmerman et al., 1992; Greene and Miller, 1996; Muretta, 2005). Self-efficacy and its influences have been demonstrated to impact the musical performance and achievement of music students (Ritchie and Williamon, 2012; Zelenak, 2019).

The importance of self-efficacy and its relationship with resilience was supported by Nowicki (2008) where social support, a sense of belonging, and self-efficacy predicted 25% of adolescents’ resilience. Sagone et al. (2020) found both that 11–19 years old became more competent as age increased and those with higher self-efficacy were more able to engage with unfamiliar situations and adapt their behavior to suit new circumstances.

In adult life, Djourova et al. (2020) explored the interrelationship of self-efficacy, resilience, and wellbeing in the professional workplace with social services workers, and found resilience mediated self-efficacy and wellbeing in relation to a person’s leadership qualities. This cocktail of self-efficacy and resilience has also been highlighted as important for developing entrepreneurship within business and industries (Hallak et al., 2018). Within education, Hamill (2003) favored self-efficacy over other factors related to resilience and showed it to be an important factor in distinguishing resilience in 16–19-years-old high school students. Resilience is strengthened by enhancing and developing protective factors such as self-efficacy, emotional intelligence, and problem-solving skills when facing adverse situations (Reivich and Shatté, 2002; Pooley and Cohen, 2010). Speight (2009) found empirical evidence of the relationship of academic self-efficacy to resilience, and Keye and Pidgeon (2013) also highlighted the importance of self-efficacy in relation to resilience with their investigation of 141 university students. McLafferty et al. (2012) reported that emotional intelligence related to resilience when studying students’ ability to cope in higher education.

### Aims

This study aims to investigate how university music student’s preparation for assessment was impacted by the forced use of remote teaching and the sudden change in assessment, as required due to Lockdown in the spring of 2020, by considering their independent practice methods (self-regulated learning strategies) to prepare for assessments, self-efficacy beliefs, and questioning their decision-making processes. It was anticipated that those with lower self-efficacy would be less strategic and might attempt to avoid the assessment task by deferring to some point in the future.

## Materials and Methods

### Context for This Research

Lectures, music lessons, and workshops all take place in dedicated spaces that are fit for purpose. Student music assessments are carried out in adequately equipped rooms, with appropriate instruments such as grand pianos or sound equipment as needed. Technicians are on hand to assist with both physical set-up and sound needs, if for example someone is using a microphone while performing a jazz song with a band. Due to the Lockdown, all teaching and assessment was moved online with a few days’ notice.

This imposed situation was highly unusual at the university, as there are established quality assurance procedures for changing aspects of a published assessment descriptor (UOC, 2020a). The “Minor Change” process involves drafting documentation that is viewed and discussed by student representatives at a bi-annual subject board meeting, as well as being reviewed by the course’s external examiner. It is then sent to the Academic Standards Committee for approval. All of this must be done in the previous academic year, so in essence, there are no surprises; changes are both planned and publicized well in advance. The only unforeseen changes that are allowed are Mitigating Circumstances which allow individuals with extraordinary and documented extenuating circumstances to have extra time or perhaps a modified assessment in order to accommodate their particular situation (UOC, 2020b).

Teachers moved their sessions online, although because of audio limitations, neither group or ensemble work nor accompanied musical performance, requiring multiple people to produce sound simultaneously, was possible to carry out in a simultaneous manner with video conferencing technology. Therefore, interactive musical work, especially in settings where a teacher might accompany their student, could not be practically achieved in the same way as a live situation. Adaptations from both the student and teacher were required to meet the demands of the situation. With forced remote learning, students needed to adopt more self-regulated learning strategies, taking initiative and carrying out the required methods and behaviors to learn and complete tasks (a detailed explanation of musical self-regulated learning strategies and behaviors, see Miksza, 2012; McPherson et al., 2019). In order not to disadvantage students, they were all given the option of either adapting to submit their performance assessments via video recording, made by the students wherever they were (e.g., their home setting) or to defer the assessment until the following semester in the next academic year with the aim of performing under the original, live setting at the university.

### Participants

Undergraduate students studying music volunteered to take part in the research. Of the 84 students, 68 identified as completely female on the 11-point gender scale (Cervone et al., 2020; Haupert, 2019), and 13 identified as completely male, two identified in the middle of the gender scale (choosing 5) and one person reported not identifying on the scale. Students represented a range of year groups, with 48 (57.1%) first year, 25 (29.8%) second year, 8 (9.5%) third year, and 3 (3.6%) fourth year students. All students were preparing for final assessments at the time the questionnaires were completed.

### Materials

This study used a combination of established questionnaires to collect empirical data and free text responses to collect qualitative data relating to student’s experiences and perspectives on their assessment during Lockdown. The questionnaire pack included the Self-efficacy for Performing scale (Ritchie and Williamon, 2011), Warwick-Edinburgh Mental Well Being short scale (WEMWBS; Stewart-Brown et al., 2009) and the Resilience scale (Cooper et al., 2013).

The self-efficacy questionnaire, originally validated by Ritchie and Williamon (2011) has been used to assess the construct widely with music students, with direct relation to performance, and more widely with regard to the wider lives of musicians (Ritchie and Williamon, 2012; González et al., 2018; Zarza-Alzugaray et al., 2020). The Self-efficacy for Performing questionnaire consists of 9 items, several of which are reverse coded. To minimize bias the self-efficacy scale were labeled with the non-specific title of “attitudes toward learning/performance activities,” as opposed to identifying the scale as measuring “self-efficacy,” as suggested by Bandura (2006).

The WEMWBS was validated by both Tennant et al. (2007) and Stewart-Brown et al. (2009), and was recently successfully used in 4 years longitudinal study of 483 musicians from 10 conservatoires in the United Kingdom (Araújo et al., 2017). The short WEMWBS wellbeing scale consists of 7 statements about feelings and mental state, each rated on a 5-point scale.

The Cooper et al. (2013) Resilience scale has been used in professional contexts, and Cooper has been consistently associated with the interaction of resilience and wellbeing (Cooper and Leiter, 2017). The scale contains 16 items that are rated on a 7-point Likert-type scale labeled strongly disagree, disagree, neither disagree nor agree, agree, and strongly agree, and addresses four components of resilience: confidence, social support, adaptability, purposefulness. These areas highlighted by these subscales have been shown to relate to aspects of resilience across people’s lives (see Masten and Wright, 2010). The Cooper et al. (2013) Resilience questionnaire used in the current research study was designed for use with people in their workplace, and as a goal of higher education is to progressively prepare students to be professionals, this questionnaire was deemed suitable to show these particular aspects of resilience in our students.

Students were asked to predict their grade on the performance assessment in question.

A phenomenological approach was adopted to collect qualitative data, following Creswell et al. (2007) and Turner (2010), and students were asked to comment, providing reasons for their choice of either submitting their performance assessment on the published date (by video during Lockdown) or deferring their assessment until the following semester (after Lockdown) and they were asked to explain how they prepared for this assessment. The full questionnaire is available from the corresponding author.

### Procedure

An email was sent to students in the department, during the Lockdown period in May 2020 (2–4 weeks before their original assessment deadlines), inviting them to complete the questionnaires for this study online.

#### Data Analysis

Statistical analysis was carried out with data collected from the questionnaires using jamovi, 2020 (version 1.2.16; 2020). Initially, data for each observed variable were screened for their appropriateness to undergo parametric testing. For this, tests of univariate normality were employed, testing for acceptable skewness values (<2) and kurtosis ratios (<4) (Kline, 1998; Fallowfield et al., 2005). Skewness and kurtosis for all quantitative measures met criteria for normality, and the normal distribution of the data was then verified through the Shapiro Wilk test (Shapiro and Wilk, 1965; Cohen, 1977). See Table 1 for descriptive statistics of these normality tests. As a result of satisfying these criterion, parametric tests were employed for all subsequent analysis. Before carrying out any relational analysis, Cronbach (1951) alpha was used to assess the reliability of all measures to test their suitability for use with this sample. A *p*-value of less than 0.05, was considered statistically significant for all analysis (Field, 2013).

**TABLE 1:** Descriptive statistics and normality tests.

	Mean (*SD, SE*)	Skewness (*SE*)	Kurtosis (*SE*)	Shapiro-Wilk (*p*)
Self-Efficacy	44.7 (8.58, 0.936)	−0.271 (0.263)	0.085 (0.520)	0.287
Wellbeing	23.1 (5.44, 0.593)	−0.242 (0.263)	0.220 (0.520)	0.623
Resilience	54.7 (7.96, 0.869)	−0.215 (0.263)	−0.232 (0.520)	0.211
Predicted mark	61.2 (11.4, 1.23)	−0.483 (0.261)	0.939 (0.517)	0.026

**SD*. *Standard Deviation; SE*, *Standard Error.**

Reliability analyses using Cronbach (1951) alpha scores were carried out for all the measures to test their suitability for use with this sample. All three questionnaires used yielded robust Alphas: The Self-efficacy for Performing scale yielded α = 0.829; the Resilience scale, comprising 16 items, yielded α = 0.776; and the WEMWBS questionnaire was very reliable, α = 0.827, signifying these measures were suitable to use with this sample.

Free text responses provided by students about their decision making and preparation for assessment were separately coded by two researchers for descriptive purposes. A thematic approach, following Braun and Clarke (2006) was used to group the reasons for the choice to submit/defer, with each condition having its own categories as drawn from the word choices used in the free text responses.

## Results

Descriptive statistics for all participants are shown in Table 1, and Table 2 presents these for groups, based on whether students chose to submit on time or to defer the assessment.

**TABLE 2:** Descriptive statistics by group: submitting now/deferring.

	Submit now: Mean (*SD, SE*)	Defer: Mean (*SD, SE*)
Self-Efficacy	46.0 (7.53, 0.934)	40.0 (10.4, 2.40)
Wellbeing	23.3 (5.40, 0.670)	22.5 (5.68, 1.30)
Resilience	55.4 (7.50, 0.930)	52.5 (9.27, 2.13)
Predicted mark	62.0 (10.1, 1.26)	57.2 (13.5, 3.10)

**SD*, *Standard Deviation; SE*, *Standard Error.**

### Quantitative Data

65 students (77.4%) reported they would embrace the alternative assessment method and submit their performance work on time as a video, filmed during Lockdown and 19 students (22.6%) chose to defer the assessment to the following academic year. An independent samples *t*-test revealed students choosing to submit instead of defer the assessment had higher self-efficacy, *t*(82) = 2.758, *p* < 0.01. There were no significant differences between resilience or wellbeing scores for the two groups of students.

In line with expectations, student self-efficacy scores positively correlated with wellbeing (*r* = 0.324, *p* < 0.01), resilience (*r* = 0.467, *p* < 0.001) and student’s predicted marks (*r* = 0.606, *p* < 0.001). Further, resilience was also correlated with wellbeing (*r* = 0.528, *p* < 0.001) and predicted marks (*r* = 0.313, *p* < 0.01).

### Qualitative Data

#### Students Choosing to Defer

Those who chose to defer clearly listed reasons having to do with the inability to successfully deliver their performances during Lockdown. Table 3 presents the categories of “live,” wanting to perform to a live audience; “resources,” a lack of physical resources; “teaching,” feeling there was an inadequate level of teaching support for musical performance with online delivery; and “COVID,” stress resulting directly from the virus itself. Representative student responses are provided to illustrate each category.

**TABLE 3:** Reasons provided for students choosing to defer their assessment*.

Category	Number	Sample response
Live	8	I am deferring my dissertation recital as I have spent months preparing it and would like an audience to share in it
Resources	7	I did not have a backing track available to me when making the decision…
Teaching	2	… and I do not feel that working on my pieces over zoom has given me enough confidence to submit them now
COVID	3	Due to anxiety surrounding the epidemic I’m finding it really hard to practice my flute (as well as complete assignments). Especially with a lack of accompaniment and teacher for support

* Two people specifically cited two categories, an example is presented in the quotation for resources and teaching; this is the response of one person.

One third of the students who chose to defer their assessment demonstrated a reduced engagement in preparing for the assessment. Those who did state they continued to practice named particular strategies, for example “self-recording,” “repeating to learn,” and “focusing on muscle memory.” Interestingly, the sample response provided which demonstrates considerable strategic engagement and teacher support (see Table 4, Practice), was from the same student who chose to defer because they felt online teaching, although they engaged with it, did not adequately prepare them for the assessment (see sample response in Table 3, Teaching).

**TABLE 4:** Assessment preparation strategies for students choosing to defer.

Category	number	Sample response
Practice	12	I have had 5 zoom singing lessons and have been doing the exercises I have been set as well as sending my teacher videos of my songs for feedback
Practice less	3	I am practicing the material less frequently as I have a lot longer to wait. I also do not have access to an acoustic piano and practice on a keyboard, I’m told, is pretty ineffective for classical repertoire and may lead to bad habits that are not possible to replicate on a Steinway
Not practicing	1	I’ve deferred so I’m not currently preparing for it
Non-musical methods	3	I am now just looking over the scores/arrangements until a new date is set, at which point I’ll begin my 1-1 lessons again. I am working nights so am unable to sing during the day at home

#### Students Choosing to Submit on the Published Deadline

Those who chose to submit in alignment with the originally published deadline provided a range of reasons that, again, clearly fell into natural grouping categories. Students wanted “to complete” saying they wanted to “get it done,” with final year students citing their desire to graduate as a driving reason to complete the assessment by the originally published date in the current academic year (see Table 5). Continuing students wanted to “avoid stress” as they progressed onto the next year of their degree, demonstrating elements of thinking ahead. Some students stated that they were sufficiently “prepared” and thus opted to submit now. Interestingly, five students felt they had no choice to defer, and were “forced” to complete now. It is unclear why they were under that impression as all students at the university had the option to defer to another semester, whether in this department or studying another subject. Two students were either unsure or had reasons completely unrelated to Lockdown for choosing to defer. Three people cited multiple reasons for their decision.

**TABLE 5:** Reasons provided for students choosing to submit their assessment by the originally published deadline*.

Category	Number	Sample response
Complete	37	Easier to get it done and out of the way, deferring it will delay it and won’t leave my mind causing later stres.
Avoid stress	13	…and worry and the uncertainty of the times at the moment could mean it may be delayed even further
Prepared	9	Preferred to complete my assessment having worked on it for the year, as I felt well-prepared
Forced	5	We don’t have a choice
Other/Unsure	2	Upcoming surgery

*Three people cited two categories. The response in Complete and Avoid stress is a quotation from one person.

The engagement with strategies was far more diverse and richer with the students choosing to pursue the original date under Lockdown conditions. One third of the students did not comment on their performance work, but listed strategies for accompanying written work. Only three students cited limiting factors in relation to their performance preparation. Unlike those who chose to defer, many of these students included several different strategies within their responses (see Table 6).

**TABLE 6:** Assessment preparation strategies for students choosing to submit by the originally published deadline.

Strategies	Number	Example responses
No comment provided about performance	14*	By researching and thinking about myself
Limitations	3	I am preparing by practicing in my bedroom but not as much as I live in terrace housing and my neighbors have been complaining
Proactive strategies:	47	Planning ahead and doing it 2 weeks before I sent mock recordings to my various tutors, and used facetime and skype to discuss areas of improvement
Planning, self-recording, seeking feedback from teachers and peers, using family to assist, engaging with new technology/methods (online lessons/self-editing), engaging with online resources (teacher videos)		Recording in advance so that I don’t have the worry of uploading. Also practicing when I can, wherever I can alongside getting my family to help me record

*12 students commented on preparation not for performance, but for accompanying written work, 1 student reported already having submitted the work at the time of completing the questionnaire, and 1 person changed their mode of assessment to no longer include performance.

## Discussion

This Lockdown period presented a unique, unprecedented situation for academia, with a sudden, dramatic shift in teaching, learning, and assessment that was outside the normal remit of expectations within the context of higher education. In this study we were able to capture both snapshots of empirical data to illustrate the relationships of student beliefs as they experienced this forced change, as well as gathering free text responses to explain their decision making and approach to their chosen timing of the assessment.

The results demonstrated correlations between self-efficacy and wellbeing, resilience, and wellbeing. Typically, one would expect someone with high self-efficacy to also demonstrate qualities of resilience. In order to have self-efficacy beliefs, one must understand the criteria for the task and have an awareness of the processes involved in achieving it. Having strong self-efficacy beliefs reflects aspects of strength, adaptability, and the capability to navigate unknowns, which directly relate to resilience and existing self-efficacy literature has repeatedly shown that self-efficacious people engage in more cognitive strategies, persist longer, and are less likely to quit in the face of adversity (Zimmerman, 2000; Urdan and Pajares, 2006). These all align with the concept of resilience (Keye and Pidgeon, 2013). It makes logical sense, therefore, that those with higher self-efficacy for performing, would also demonstrate more strategies as they worked toward that achievement.

Students who adhered to the published dates for assessment despite the Lockdown conditions responded with a sense of “must.” Although these students choose to submit on time, the task was still significantly different, as they had to perform wherever they were, organize and achieve an appropriate recording, and deliver this to the teacher in the required manner. These students had higher self-efficacy for the task in question than those who chose to defer, but their reported need to “get it done” to either allow for graduation or to avoid further stress demonstrates the impact of this unique Lockdown situation.

Although those who chose to defer cited reasons why they could not complete their assessments on time, this was not a considered decision resulting from simply embracing a few extra months to learn, but instead was a statement of “can’t,” expressing unsurpassable constraints. When “can’t” becomes a mindset, the possibility of achievement is removed. This perspective of having an unsurmountable challenge in progress or achievement also aligns with someone who has lower self-efficacy beliefs (Muretta, 2004; Speight, 2009). These students were more articulate with their reasons why *not.* There were a very few in this group who stated strategic engagement beyond continuing to “practice.” The time delay and uncertainty of a future assessment that would take place in the next academic year meant that students did not necessarily perceive a hierarchical path to the goal. The clarity of the proximal goal they had been approaching was taken from them, along with the accompanying equipment and support. This has implications for educators when considering how to provision appropriate student support for the task. The goal posts have moved in a way that is new for everyone.

Despite the dramatically altered conditions where teaching was forced online, and where students were without the physical rehearsal space or support and had to learn the technological skills of recording themselves, students did demonstrate a variety of strategic approaches, which are qualities associated with self-efficacy, as they pursued submitting their recorded assessments on time (Cervone et al., 2004). The multiplicity of strategies and the use of self-directed learning to engage with resources, people, and to develop new skills aligns with expected behavior of people with higher self-efficacy beliefs.

Strategic use, and in this case self-regulation involves actively initiating learning and negotiating challenges through choice and strategy use as opposed to following set, external instruction (Ritchie and Williamon, 2013). The strategies students adopt depend on their resourcefulness and capability for engaging with cognitive processes; if students either do not understand or are not proficient with a method, they are unlikely to use it. Although self-regulation implies the student undertake these processes independently, within higher education students are not always aware of being independent agents (Scott et al., 2015; Henri et al., 2018).

Any assessment task will be benchmarked and should be aligned with the taught skill set (Biggs and Tang, 2015). This unforeseen shift in the student experience has highlighted an opportunity to indeed support students in areas of skill development, awareness, and indeed resilience. Many of these students did demonstrate strategic thinking and independent learning strategies, but it was clear that there were also negative motivators, and it should not be out of avoidance of adverse consequences that students are motivated to complete their assessments. As blended forms of learning become the “new normal” in the future, and include alternate forms of engagement and assessment, educators should take a broader view of the way they prepare and expect students to engage with their curricula.

## Limitations and Future Directions

Previous music research has not been undertaken with a similar situation, and to recreate these Lockdown conditions in future studies to replicate the inverse relationship of resilience to self-efficacy and wellbeing would require imposing significant restrictions on student learning and might be difficult to ethically rationalize recreating.

Our findings provide further insight into the influence of COVID-19 on student experience. However, given the scarcity of research including musicians during this period (Philippe et al., 2020; Spiro et al., 2020; Tymoszuk et al., 2021), the sample size being determined by accessibility and time constraints (as is common in psychology research; Lakens, 2021) and the influence posed by this pandemic, we encourage caution when interpreting the study’s *p*-values in a generalizable way. As such, future researchers must extend the current findings (e.g., comparing the impact of teaching and student experience before, during and after COVID-19).

There is a need for self-efficacy, wellbeing, and resilience in our changing world, and specifically for music students as they prepare to enter professional careers. The present research demonstrates the interrelatedness of these constructs and the importance of a wider awareness of the student, beyond providing material and access options, to include understanding aspects of the student’s experience of learning and their self-beliefs. As music educators we can equip students with both strategic methods for self-regulation and develop resilience through dedicated training programs to enable them to better cope, not only in their educational environment and after graduation in their future workplace, and facilitate adaptability when unforeseen circumstances force change.

## Data Availability Statement

The datasets presented in this study can be found in online repositories. The names of the repository/repositories and accession number(s) can be found below: https://osf.io/n2up6/.

## Ethics Statement

The studies involving human participants were reviewed and approved by the University of Chichester Ethics Committee. The patients/participants provided their written informed consent to participate in this study.

## Author Contributions

LR contributes to the concept, design, definition of intellectual content, data analysis, manuscript preparation, and the manuscript editing. BS contributes to the design, data analysis, manuscript preparation, and the manuscript editing. Both authors contributed to the article and approved the submitted version.

## Conflict of Interest

The authors declare that the research was conducted in the absence of any commercial or financial relationships that could be construed as a potential conflict of interest.
